# Synthesis, Functionalization and Applications of Nanocarbons

**DOI:** 10.3390/nano12162738

**Published:** 2022-08-10

**Authors:** Simona Bettini, Gabriele Giancane

**Affiliations:** 1 Department of Biological and Environmental Sciences and Technologies, DISTEBA, University of Salento, Via per Arnesano, I-73100 Lecce, Italy; 2 Department of Cultural Heritage, University of Salento, Via Birago 64, I-73100 Lecce, Italy

The Special Issue “Synthesis, Functionalization and Applications of Nanocarbons” starts from the growing interest of the scientific community in carbon-based materials and the various applications of these versatile compounds. Among the first allotropic forms of carbon that strongly attracted the attention of scientists, there are the fullerenes; then, several works were produced with carbon nanotubes; and, in 2010, studies about graphene earned the Nobel prize for Andre Geim and Konstantin Novoselov. More recently, other carbon-based materials such as nanodiamonds, onion-like carbon nanomaterials, carbon dots, and carbon quantum dots were added to the large and fascinating family of carbon-based nanomaterials.

The Special Issue presents seven contributions focused on carbon-based nanostructures in the form of graphene, carbon dots (CDs), nanodiamonds, onion-like carbon nanomaterials and carbonous doped materials. The applications of nanocarbons include wearable devices, water remediation, energetic applications and the fabrication of sensing devices. The use of carbon nanostructures in wearable devices allowed the fabrication of highly stretchable materials, preserving their electric properties and ensuring high biocompatibility [1]. Co- and N-doped carbon-based nanostructures were synthetized by Gomez-Lopez and co-workers by means of a new and ecofriendly solvent-free process [2]. The obtained materials were proposed as electro-catalysts for energetic and environmental applications.

An environmentally sustainable procedure to obtain graphene oxide starting from graphene was proposed by C. F. Costa and collaborators [3]. An alternative method to obtain onion-like carbon nanostructures starting from detonation nanodiamonds was proposed in [4]: an ethanol-dispersed hydrogenated nanodiamonds suspension was irradiated by means of a green pulsed laser and the chemical–physical features of the obtained nanostructures were critically evaluated by the authors. Li and Zhou studied the effect, in terms of deformations and stress, of localized heating obtained by carbide-bounded graphene on polymeric and vitreous materials [5].

Finally, a research article and a fascinating review concerning a new emerging class of carbon-derived materials, carbon dots, are presented in this Special Issue [6,7]. Several synthetic methods are examined, as well as doping materials used for tuning and for modifying the properties of CDs. Methods of characterizing CDs are reported by Sousa et al., highlighting the most relevant physical and chemical characteristics of carbon dots. In conclusion, examples of the application of CDs in sensing, energy harvesting and conversion, optoelectronics and photocatalysis are reported.
